# Case report: A case of advanced gastric cancer with multiple skin metastases, with significant relief from immunotherapy

**DOI:** 10.3389/fimmu.2024.1356350

**Published:** 2024-02-29

**Authors:** Wen Hao, Ruimin Chang, Jingxin Liu, Yibing Wang, Meijin Ren, Kai Xin, Baorui Liu, Jiaqi Xie, Yang Yang

**Affiliations:** ^1^ Department of Oncology, Nanjing Drum Tower Hospital, Drum Tower Hospital Clinical College, Nanjing University of Chinese Medicine, Nanjing, Jiangsu, China; ^2^ Nanjing Drum Tower Hospital, Affiliated Hospital of Medical School, Nanjing University, Nanjing, China

**Keywords:** gastric cancer, cutaneous metastasis, immunotherapy, genetic sequencing, PD-1

## Abstract

Gastric cancer is the fifth leading cause of cancer-related mortality worldwide, with a low 5-year survival rate in advanced stages. Cutaneous metastasis is rare in gastric cancer, with only 0.8-1% incidence. We reported a rare case of female gastric cancer. The patient had undergone subtotal gastrectomy and chemotherapy 13 years ago, followed by a subsequent surgery of residual stomach, partial jejunum, and partial colon resection 11 years later. The pathological examination revealed poorly differentiated stomach adenocarcinoma, Lauren classification: diffuse type. The patient received 2 cycles of SOX chemotherapy. Two years later, cauliflower-like skin nodules, which were surgically excised, appeared on the back. The histopathological examination showed a spindle cell tumor; no specific anti-tumor treatment was administered. Six months later, the skin lesions increased in size and number, spreading to the neck, chest, and abdomen, presenting as erythematous patches with some cauliflower-like elevations. A skin biopsy of a 1cm0.5cm0.3cm lesion on the left abdomen was performed, and based on the immunohistochemistry, clinical history, and the possibility of metastatic or infiltrating adenocarcinoma, the gastrointestinal origin was highly suspected. Genetic testing was performed on the gastric recurrence and skin lesions, revealing 103 shared genetic variations, further suggesting the skin metastasis originated from gastric cancer. Subsequently, the patient received 10 cycles of immunotherapy combined with intravenous chemotherapy (200mg Tislelizumab and 100mg albumin-bound paclitaxel). The treatment response was evaluated as partial remission, with significant improvement in the skin lesions compared to before. This case highlights the possibility of tumor metastasis in patients with extensive skin lesions in advanced gastric cancer. Early examination, diagnosis, skin biopsy, immunohistochemistry, and genetic sequencing are recommended.

## 1 Introduction

Gastric cancer is the fifth leading cause of cancer-related deaths worldwide, with a particularly low 5-year survival rate for advanced gastric cancer (1). Lymph nodes, liver, and peritoneum are common sites of recurrence and metastasis in gastric cancer, while skin metastasis is rare, occurring at a rate of only 0.8-1% (2). Skin metastasis in gastric cancer mainly occurs through hematogenous and lymphatic spread, and its mechanism is complex and not yet fully understood. Cancer cells separate from the primary tumor, invade and infiltrate the blood or lymphatic vessels, survive, invade host tissues, and proliferate at distant sites. Skin metastasis occurs in up to 10% of patients with visceral malignancies (3). Skin metastasis in gastric cancer is more common in males. It typically appears several months or years after the initial diagnosis but can also be the initial sign of the first diagnosis or recurrence (4). Skin metastasis in gastric cancer usually occurs on the abdominal skin (5, 6). It presents as solitary or multiple painless nodules with a red or purple color in the dermis or subcutaneous tissue. Occasionally, they may be arranged in a linear pattern (7). It can also manifest as cellulitis or erysipelas-like erythematous plaques (erysipelas carcinoma) (5, 8), scar alopecia (8), or cauliflower-like growths and ulcers (9). According to literature reports, the average survival period after the occurrence of skin metastasis is approximately 3 months. There is limited literature available on gastric cancer with skin metastasis.

In this article, we report a rare case of a female patient with gastric cancer who developed extensive skin lesions after gastric cancer recurrence surgery. After receiving immunotherapy combined with chemotherapy, the skin lesions improved significantly compared to before, and the patient has survived for over 1 year.

## 2 Case report

The patient is a 61-year-old female who presented with erythematous changes in the skin of the back, neck, chest, and abdomen, some of which had cauliflower-like elevations. She received treatment at the Oncology Treatment Center of Nanjing Drum Tower Hospital in Nanjing in July 2022. In 2009, she underwent a subtotal gastrectomy at an external hospital and received four cycles of postoperative intravenous chemotherapy. In April 2020, the patient visited the Traditional Chinese Medicine Hospital in Pukou District, Nanjing, due to discomfort in the upper abdomen for 2 months. Gastroscopy showed “residual gastric cancer,” and she underwent “residual stomach” + “partial jejunum” + “partial colon resection” surgery. A biopsy on the surgical specimen revealed poorly differentiated adenocarcinoma of the residual stomach, Lauren classification: diffuse type. From June 8, 2020, the patient received two cycles of SOX chemotherapy (oxaliplatin 150mg, tegafur 50mg in the morning and 75mg in the evening, orally, on days 1-14) at the Traditional Chinese Medicine Hospital in Pukou District, Nanjing. Regular follow-up examinations were conducted after chemotherapy. In September 2021, the patient visited the Central Hospital in Pukou District, Nanjing, due to the appearance of cauliflower-like masses on the back skin for 3 months without any apparent cause. Surgical excision was performed, and the pathological result of the back mass showed squamous cell carcinoma. No specific anti-tumor treatment was administered thereafter. From March 2022, the patient’s skin lesions increased in size and number, spreading to the skin of the neck, chest, and abdomen, presenting as erythematous changes, some with cauliflower-like elevations. Finally, she came to our hospital for treatment.

**Figure 1:**
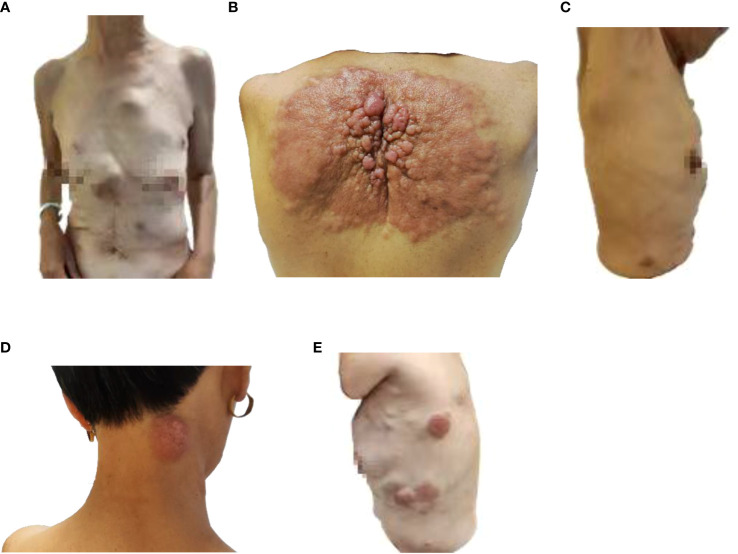
Image of skin lesions on August 9, 2022. **(A)** Front view of the patient, **(B)** Back view of the patient, **(C)** Right side view of the patient, **(D)** Neck view of the patient, **(E)** left side view of the patient.

**Figure 2:**
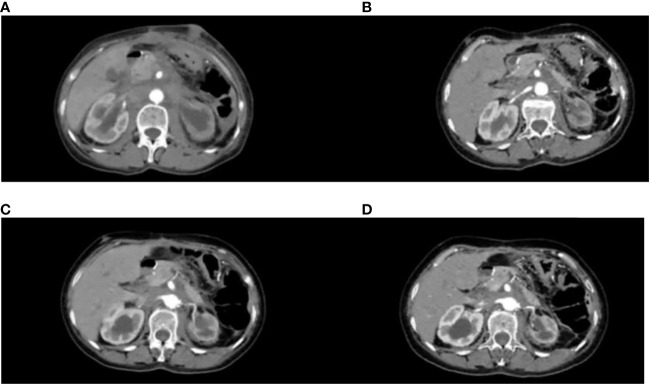
CT variation diagram. **(A)** Patient 2022.8.8 CT image, **(B)** Patient 2022.10.27 CT image, **(C)** Patient 2023.3.29 CT image, **(D)** Patient 2023.6.5 CT image.

**Figure 3:**
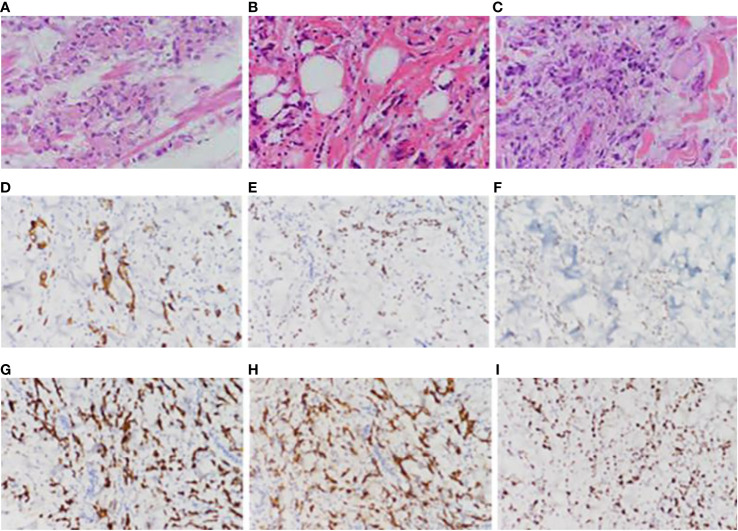
Pathological picture. **(A)** Pathological images of the patient’s remnant stomach, partial jejunum, and partial resection specimen taken on April 30, 2020, **(B)** pathological images of the back skin lump taken on October 25, 2021, **(C)** pathological images of the left abdominal skin tissue taken on July 20, 2022, **(D)** immunohistochemical CK7 pathological images of the left abdominal skin tissue taken on July 20, 2022, **(E)** immunohistochemical CDX-2 pathological images of the left abdominal skin tissue taken on July 20, 2022, **(F)** Immunohistochemical STAB2 pathological image of left abdominal skin tissue taken on July 20, 2022, **(G)** Immunohistochemical CK8-18 pathological image of left abdominal skin tissue taken on July 20, 2022, **(H)** Immunohistochemical Villin pathological image of left abdominal skin tissue taken on July 20, 2022, **(I)** Immunohistochemical ki67 pathological image of left abdominal skin tissue taken on July 20, 2022.

Partial areas of erythematous changes and cauliflower-like raised masses are observed on the skin of the patient’s neck, chest, and back. There is no increased skin temperature, obvious ulceration, erosion, or exudation. Upon palpation, the masses fluctuate, accompanied by tenderness and pruritus (**Figure 1**). Imaging examination reveals fluid accumulation in the abdominal and pelvic cavities, indistinct fat planes within the abdominal cavity, multiple soft tissue masses behind the peritoneum, unclear visualization of the inferior vena cava, thickening of the colonic hepatic flexure wall with luminal narrowing, indistinct mesenteric fat planes with multiple nodular opacities, and multiple soft tissue nodules in the subcutaneous tissue and muscles of the abdominal wall with local skin thickening (**Figure 2A**). On July 20, 2022, a skin tissue sample was obtained from the left abdomen at Drum Tower Hospital for pathological biopsy, which revealed infiltrative growth of atypical cells in the skin tissue, subcutaneous layer, and adipose tissue of the left abdomen. Immunohistochemical analysis showed that the sample was consistent with metastatic or infiltrative adenocarcinoma, with a high possibility of gastrointestinal origin (**Figures 3A-I**). Immunohistochemical testing results showed that the tumor cells expressed CK7 (++), CK20 (-), Villin (+++), Ki67 (approximately 70%+), CDX-2 (+++), SATB2 (+), CK8/18 (+++), Mammaglobin (-), GCDFP15 (-), and AR (-).

Specimens of the residual stomach, partial jejunum, and partial resection obtained on April 30, 2020 (**Figure 3A**), and a skin mass specimen from the back obtained on October 25, 2021 (**Figure 3B**), were further analyzed in the Pathology Department of Drum Tower Hospital, Nanjing, on June 16, 2023. The results revealed a low-adhesive adenocarcinoma with a high possibility of gastrointestinal origin. The residual stomach, partial jejunum, and partial resection specimens obtained at Pukou Traditional Chinese Medicine Hospital in 2020, as well as the left abdominal skin tissue sample obtained at Drum Tower Hospital on July 20, 2022, were sent to Shanghai Ben Medical Laboratory Co., Ltd. for whole-exome sequencing (WES) genetic testing. The testing results showed 155 gene mutations and 328 neoantigens in the gastric lesion, and 190 gene mutations and 385 neoantigens in the skin metastatic lesion. 103 shared mutations between the two lesions, suggesting that the skin lesion originated from gastric cancer.

**Figure 4:**
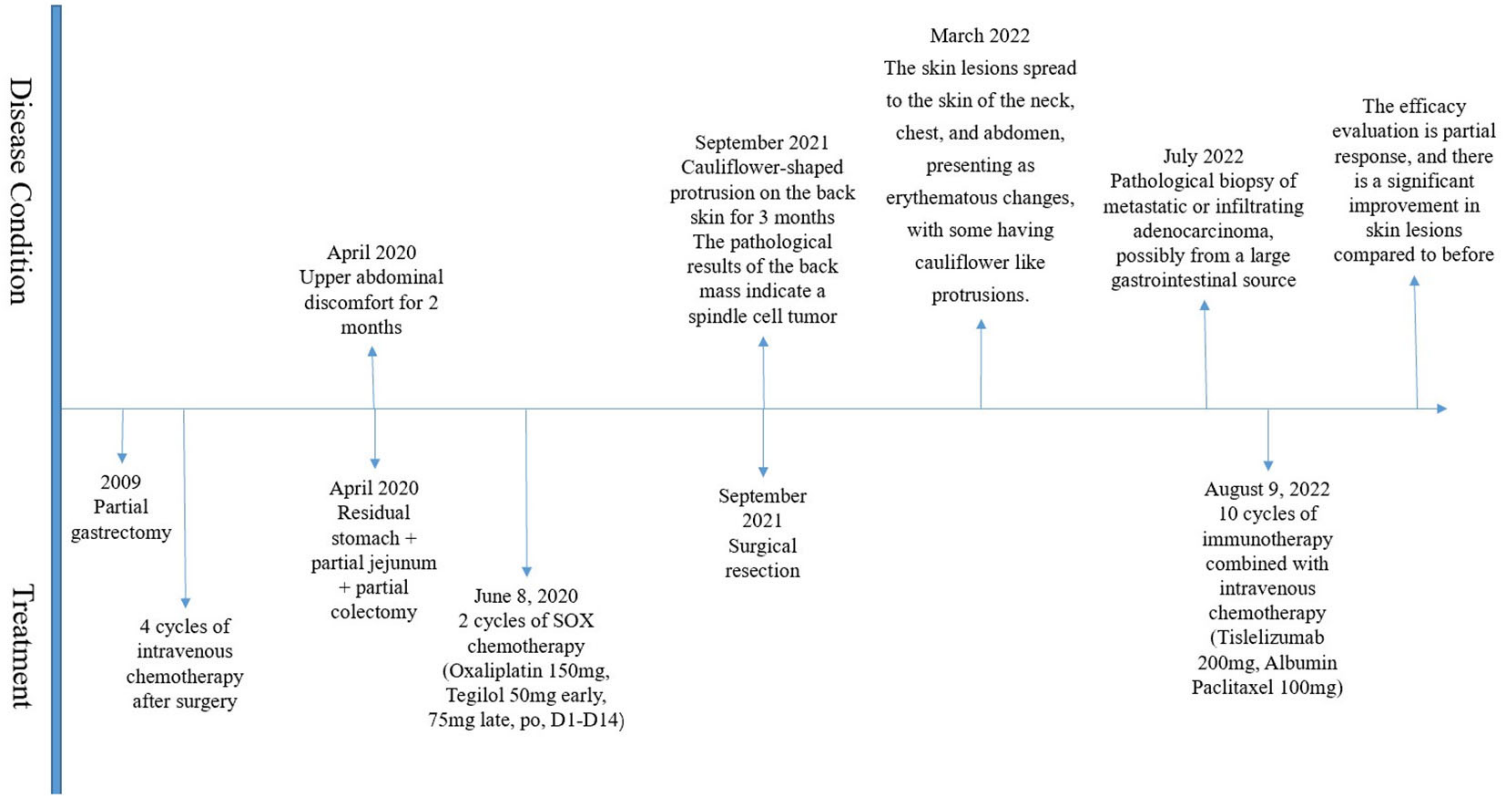
Treatment process diagram.

**Figure 5:**
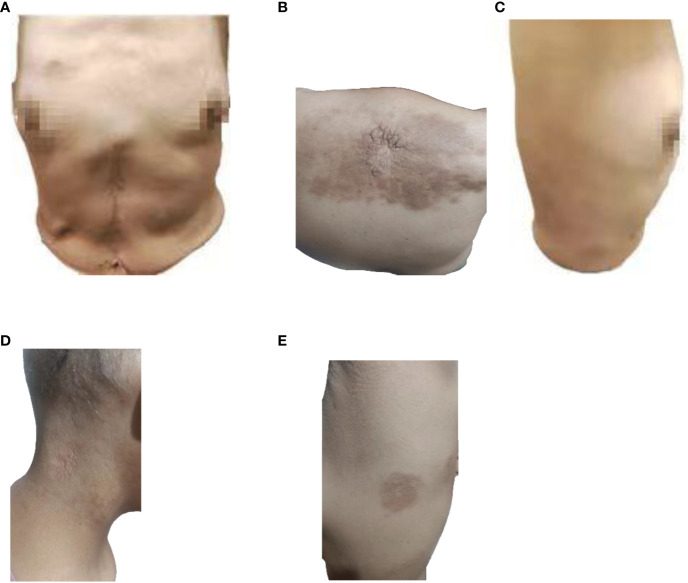
Image of skin lesions on May 4, 2023. **(A)** Front view of the patient, **(B)** Back view of the patient, **(C)** Right side view of the patient, **(D)** Neck view of the patient, **(E)** left side view of the patient.

**Figure 6:**
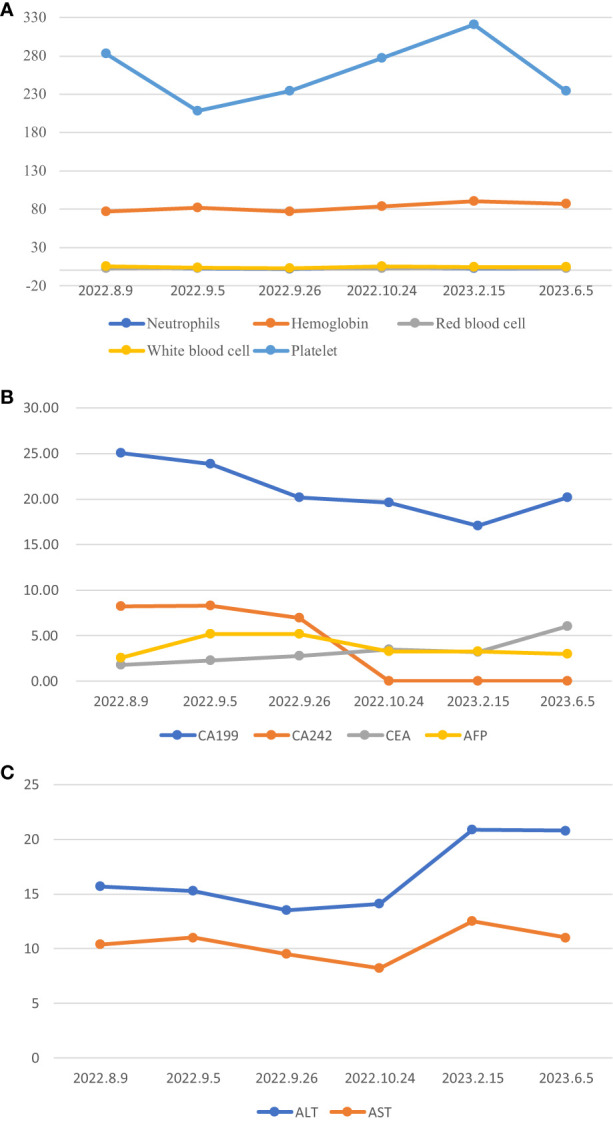
*:Normal value range for tumour markers, CA199(0-27U/ml), CA242(0-10U/ml), CEA(0-5.2ng/ml), AFP(0-7ng/ml). **(A)** Blood routine changes, **(B)** Changes in tumor markers, **(C)** Liver function change map.

The patient has been receiving chemotherapy with Bevacizumab (200mg) and Albumin-bound Paclitaxel (100mg) (**Figure 4**) at Drum Tower Hospital, Nanjing, since August 9, 2022. A total of 10 cycles have been completed, We conducted annual monitoring of patients from 9 August 2022 - 20 July 2023, and the therapeutic evaluation showed partial tumor remission (**Figure 2**). Additionally, there was a significant improvement in the patient’s skin lesions (**Figure 5**). From August 9, 2022, to June 5, 2023, the patient’s blood routine examination (**Figure 6A**) and liver function test results (**Figure 6B**) showed no significant abnormalities. The tumor markers exhibited a decreasing trend and remained within the normal range (**Figure 6C**).

The patient’s initial surgery was performed in 2009 at an outside hospital, and we were unable to locate the hospital in question to obtain specific information due to the passage of time and the fact that the patient and his family did not keep the relevant data as well as information about the hospital visit. Meanwhile, our country’s preservation of follow-up data and basic epidemiological hygiene needs to be improved and learnt from European and American countries. Therefore there was no information about the stage of the disease, the extent of spread, regional lymph node status, tumor marker levels before and after gastrectomy, details of the surgery, and the histological structure of the resected tumor after the initial surgery. However, imaging specialists and pathologists can judge that the patient belonged to the total gastrectomy Roux-en-y jejunostomy and D2 radical surgery based on the number of anastomotic nails and the position of the anastomosis shown on the CT taken since the patient’s admission to the hospitaljudge that the patient belonged to the total gastrectomy Roux-en-y jejunostomy and D2 radical surgery with spleen preservation and no recurrence for 10 years which is a relatively common surgery in East Asian countries.

## 3 Discussion

Cutaneous metastasis commonly occurs in breast cancer, lung cancer, colorectal cancer, ovarian cancer, and other tumors (10), while cutaneous metastasis of gastric cancer is extremely rare. Our review of the literature revealed: a case of a 60-year-old woman with cutaneous metastasis of gastric cancer, whose facial skin showed painless pruritic eczema, resembling acute dermatitis. She had earlier undergone a total gastrectomy for advanced gastric cancer. After 14 months, she developed eczematous facial lesions; the presumptive diagnosis was acute dermatitis. However, a skin biopsy unexpectedly revealed cutaneous metastasis of gastric cancer. After 6 months of systemic chemotherapy with capecitabine and cisplatin, the cutaneous metastasis was markedly improved and clinically complete remission was accomplished (11). Another report: the case of a 78-year-old woman with small finger-tip-sized reddish nodules in the skin of her chest wall. Her past history showed total gastrectomy for gastric cancer (T1N2MO: stage2) 17 months previously. The biopsy of the skin lesion demonstrated a metastatic skin cancer of papillary adenocarcinoma. Chemotherapy treatment with a combination of TS-1 and Paclitaxel proved ineffective, and the patient died 3 months after the diagnosis of skin metastasis (12). And another case: A 66-year-old woman presented with two masses on her scalp. A biopsy of the scalp tumors showed mucinous adenocarcinoma and CT showed tumors in the stomach. She received S-1 chemotherapy and after two cycles of chemotherapy, both the stomach and scalp tumors were reduced in size and CT showed no evidence of other metastases. Performed a total gastrectomy followed by postoperative chemotherapy with S-1. Ten years after surgery, no new lesions were found (13). There are fewer reports in the literature about gastric cancer with skin metastases, and these reports are older, compared to which the features of gastric cancer with skin metastases in our case are more typical. The pathogenesis of cutaneous metastasis is still unclear. Through genetic testing of the gastric cancer patient’s recurrent gastric lesion and cutaneous metastatic lesion, 87 gene mutations specific to the cutaneous lesion were detected. In our case database, another patient with cutaneous metastasis of gastric cancer was found, and a comparison of the genetic testing results of the cutaneous lesions in the two patients revealed mutations in PIK3CA, KMT2D, and MUC16. Further comparison of the cutaneous metastatic lesions and the primary gastric lesions revealed that PIK3CA and MUC16 gene mutations were newly acquired mutations in the cutaneous metastatic lesions, suggesting that these gene mutations may be associated with cutaneous metastasis of gastric cancer. Regarding PIK3CA gene mutations, relevant studies have shown that triple-negative primary breast cancer (BC) has a high proportion (36.4%) in tumors that develop into cutaneous metastatic tumors (CM) (14). 15% of metastatic tumor patients have changes in their tumor molecular types, and 48.5% of metastatic tumors have additional molecular changes compared to the primary tumors, with the most common being amplification of MYC and MDM4, as well as mutations in TP53 and PIK3CA. The literature review revealed limited research on PIK3CA gene mutations in gastric cancer patients with cutaneous metastasis. Whether PIK3CA gene mutations in gastric cancer patients indicate the possibility of cutaneous metastasis requires further scientific research.

Cutaneous metastasis of gastric cancer is one of the indicators of advanced-stage tumors, suggesting a poorer prognosis for patients. However, approximately 61% of patients still receive aggressive treatments such as surgery, chemotherapy, or radiation therapy (15). Immune checkpoint inhibition is a new standard of targeted therapy in the treatment of advanced or metastatic gastric cancer (GC) and is represented in various combinations with and without chemotherapy in every therapy line within clinical trials. In advanced adenocarcinoma of GC, gastroesophageal junction cancer (GEJC) and esophageal cancer (EC), the combination of nivolumab and chemotherapy in first-line therapy improves overall survival (OS) in PD-L1 (programmed cell death protein 1)-positive patients with approval in Europe (PD-L1 CPS (combined positivity score) ≥ 5), USA and Taiwan (CHECKMATE-649) and pembrolizumab plus chemotherapy for GEJC and EC in Europe (CPS ≥ 10) and the USA (KEYNOTE-590). Furthermore, pembrolizumab plus trastuzumab and chemotherapy show clear benefits in OS and are approved as first-line treatment of Her2 (human epidermal growth factor receptor-2)-positive tumors in the USA (KEYNOTE-811). Nivolumab demonstrates superior OS regardless of PD-L1 expression in third-line therapy with approval in Japan (ATTRACTION-02) and pembrolizumab prolonged the duration of response in PD-L1 positive patients with approval in the USA in PD-L1 CPS ≥ 1 patients (KEYNOTE-059) (16). Therefore, immunotherapy has brought new hope to patients with metastatic gastric cancer. Whole exome sequencing (WES) was performed on the gastric and skin samples to investigate why this patient responded significantly to immunotherapy. The results revealed 155 gene mutations in the recurrent gastric lesion, with a tumor mutational burden (TMB) of 5.1 Muts/Mb and microsatellite stability (MSS). Analysis of neoantigens identified 328 new antigens. 190 gene mutations were detected in the cutaneous metastatic lesion, with a TMB of 6.2 Muts/Mb and MSS. Analysis of neoantigens identified 385 new antigens. The proportion of new antigens to the total number of mutations in the patient’s tumor tissue was higher than that in 80% of gastric cancer patients, which may contribute to the favorable response to immunotherapy. Neoantigens are tumor-specific mutated proteins generated by genomic alterations in tumors. After processing into short peptides (epitopes), neoantigens are presented on the cell surface by major histocompatibility complex (MHC, also known as HLA in humans) molecules. The tumor cells predominantly express MHC class I (HLA A/B/C/E/F/G), which presents intracellular antigens to CD8+ T cells. T cells recognize neoantigens as “non-self” and activate an immune response. Limited clinical studies have shown that the quantity and quality of neoantigens may be associated with immunotherapy (17). In a study using neoantigen vaccines for melanoma treatment (18, 19), 10 patients were enrolled, of which 8 had a high mutation rate. Six patients received the vaccine, and at a mean follow-up of 25 months (20-32 months), 4 patients in stage III B/C showed no disease recurrence, while 2 stage IV patients experienced disease progression after the final immunization but achieved complete radiographic response after subsequent treatment with PD-1 monoclonal antibody pembrolizumab (20). This suggests that neoantigens play a crucial role in tumor immunotherapy. Neoantigens represent an ideal target in immunotherapy, and with increasing scientific and clinical evidence demonstrating the efficacy of neoantigen-based vaccine therapies in various cancer types, there is ample reason to believe that neoantigen-based vaccines will become a promising field in cancer immunotherapy (21).

This case is extremely rare and of high clinical value. When patients with a history of gastric cancer present with skin lesions, the possibility of tumor recurrence and metastasis should be highly suspected. Our case is an essential addition to understanding skin metastasis in gastric cancer, as it suggests that skin metastasis can also occur in female gastric cancer patients after surgery. Additionally, genetic testing of skin and gastric cancer lesions can aid in diagnosing and treating such patients. More neoantigens in the tumor suggest a more tremendous potential for immune therapy to have a significant impact. It helps identify gastric cancer patients who may be sensitive to immune therapy, potentially serving as a new immunotherapy biomarker. The limitation is that this is only a single case, and future research should focus on better selecting metastatic gastric cancer patients who would benefit from immune therapy, to help more patients with metastatic gastric cancer. Our case is the first reported rare case of a female gastric cancer patient with skin metastasis who has benefited from immune combination chemotherapy for nearly a year.

## Data availability statement

The original contributions presented in the study are included in the article/supplementary material. Further inquiries can be directed to the corresponding authors.

## Ethics statement

The studies involving humans were approved by Medical Ethics Committee of Drum Hospital Affiliated to Nanjing University School of Medicine. The studies were conducted in accordance with the local legislation and institutional requirements. The participants provided their written informed consent to participate in this study. Written informed consent was obtained from the individual(s) for the publication of any potentially identifiable images or data included in this article.

## Author contributions

WH: Writing – original draft, Data curation, Methodology, Visualization. YY: Writing – original draft, Data curation, Investigation. JX: Writing – review & editing. JL, RC, MR: Writing – review & editing, Supervision. KX: Supervision, Writing – review & editing, Resources. BL: Supervision, Writing – review & editing, Validation. All authors contributed to the article.
